# Synthesis and multipurpose assessment of Cu(II) and Pd(II) Schiff base complexes as catalysts and antimicrobial agents

**DOI:** 10.1038/s41598-026-47355-3

**Published:** 2026-04-19

**Authors:** Radhika Malav, Sriparna Ray

**Affiliations:** Catalytic Applications Laboratory, Department of Chemistry, School of Physical and Biological Sciences, Faculty of Science, Technology and Architecture, Manipal University Jaipur, Dehmi Kalan, Jaipur, Rajasthan 303007 India

**Keywords:** Carbon–carbon cross coupling reaction, Suzuki–Miyaura cross coupling, Schiff base, Antimicrobial activity, Degradation of organic dyes, Biochemistry, Chemistry

## Abstract

Schiff base derivatives of isatin were synthesized with tryptamine in excellent yields to explore their broad applications as ligand precursors. Further, the ligand was reacted with Cu(OAc)_2_.H_2_O and PdCl_2_(CH_3_CN)_2_ to form the corresponding complexes with a 1:2 metal-to-ligand stoichiometry. The ligand and its complexes were analyzed with various techniques, including UV, FTIR, ESI-MS spectroscopy, and some physicochemical methods. Thermal analysis displayed high stability, with the Cu(II) complex remaining stable upto 250 °C and the Pd(II) complex showed no major decomposition until 340 °C. The molecular composition was examined with the help of XRD and CHN techniques. The antimicrobial evaluations revealed that complexation significantly enhanced bioactivity compared to the free ligand. The complexes exhibited moderate to strong growth inhibition against bacterial (*E. coli*, *P. syringae*, *S. aureus*, and *S. epidermidis*) and fungal strains (*A. flavus* and *C. albicans*). Additionally, the synthesized complexes were utilized as catalysts in the Suzuki-Miyaura coupling reaction of bromobenzene and phenylboronic acid. Another catalytic proficiency in reductive degradation of common dyes, like methylene blue (MEB), Rhodamine-B (Rh-B), Congo red (CR), and methyl orange (MO), was investigated for copper and palladium complexes. Both metal complexes facilitated the reductive degradation of hazardous organic dyes, in aqueous media.

## Introduction

Transition metals and their complexes have attracted a lot of attention, as these can be employed as catalysts for a variety of organic transformations^1,2^. Amongst these transformations, the C-C coupling reactions have been researched extensively due to their various applications in designing intermediates in the synthesis of numerous agrochemicals, pharmaceuticals, and natural compounds^3^. Till date, many different sorts of catalysts have been developed to accomplish these reactions^4^. Different transition metals, including palladium, cobalt, and copper, are useful for both homogeneous and heterogeneous catalysis^5,6^. Numerous C-C bond-producing reactions like Suzuki-Miyaura coupling (SMC), Heck cross-coupling, Sonogashira reaction, etc., are facilitated by catalysts formed from these metal complexes^7,8^. In Suzuki-Miyaura reactions, which include the reaction of aryl boronic acid and aryl halides, palladium complexes have been employed as an immensely tunable catalyst^9^. Additionally, the use of non-precious, inexpensive and easily synthesized metal catalysts in cross-coupling processes is likewise in focus^10,11^. As a result, the development of Cu-based complexes that catalyzed the coupling reaction has gained more attention currently. As copper is inexpensive, due to its natural abundance, it makes possible to design cost-effective chemical transformations. These processes have been successfully carried out using metal complexes with a diversity of ligands, comprising pincer, phosphines, N-heterocyclic carbenes (NHCs), thiols, palladacycles, etc.^12–14^. In contrast to these ligands, Schiff base (SB) ligand, resulting from a condensation reaction of a primary amine and a carbonyl group, is an interesting ligand in coordination chemistry^15^. The SB ligands are inexpensive, synthesized and characterized easily. They are thermally and chemically stable and can form complexes with variety of transition metal centers^16^. SB ligands with N, O-donors along their transition metal complexes catalyzed various organic transformation reactions. Additionally, they have garnered a lot of attention due to their applicability in several industries^17^. Consequently, priority is given to using SB metal complexes in carbon-carbon bond-forming reactions. Suzuki reaction has been considered as one of the most important pathways for the preparation of biaryls. These biaryls have key structural roles in a variety of polymers, natural goods, agrochemicals, and pharmaceutical intermediates^18,19^. This cross-coupling reaction has various advantages over the other cross coupling reactions as the reaction proceeds in mild conditions, has easy availability of organoboron reagents, and has great functional group tolerance.

Another aspect of common concern is the industrial organic dyes, which are broadly used in several areas such as textiles, paper, plastic, leather, pharmaceuticals, and packaging^20,21^. Dyes discharged from industries enter water bodies and pose challenges for removal due to their complex molecular structure^22^. Even extremely low aqueous concentrations of their wastes make the water source unfavorable. Many of these dyes also induce severe toxicity in aquatic life via the slow release of harmful chemicals, causing mutagenic and carcinogenic effects^23^. Moreover, their discharge into water bodies poses severe environmental risks due to their resistance to degradation. Also, these dye pollutants can cause allergies, liver and kidney damage, skin irritation, and neural disorders in animals and humans^24^. Therefore, discharging the untreated wastewater containing organic dyes into water bodies causes widespread environmental contamination all over the ecosystem^25^. Emerging technologies in dye degradation, particularly advanced oxidation, reduction, and photocatalysis processes, offer promising solutions for the complete elimination of these pollutants^26–28^. Although different methods (biological treatment, chemical treatment, ion exchange, membrane separation, ultrafiltration, etc.) are utilized for eradicating these dyes from water sources^29,30^. The degradation/reduction process has been superior because of its advantages, such as ease of procedure. Metal complexes or metal nanoparticles are extensively used in the reduction of organic dyes as catalysts. Herein, Cu and Pd complexes have been used as a catalyst in the reduction of common dyes such as Rhodamine-B (Rh-B), methyl orange (MO), methylene blue (MEB), and Congo red (CR), which are harmful to the environment. Given the persistent nature of organic dye pollutants, research into their degradation not only addresses environmental and public concerns but also drives innovation in material science and catalytic technologies. The study aims to assess the efficiency of novel catalytic systems for the degradation of organic dyes, contributing knowledge towards the development of robust water purification strategies.

Additionally, transition metal complexes have the capability to exhibit biological activity and are being actively investigated in this area of research^31–33^. SB ligands resulting from isatin are remarkably valuable metal chelators through coordination^34^. Chelated complexes exhibit enhanced biological potentials in similar environments compared to either the ligands or the metal precursors^35,36^. Azomethine group of SBs as well as additional substituents on the ligands perform a significant role in antimicrobial activities^37^. The enhanced activity of complexes is influenced by structural aspects such as solubility and cell penetrability, which may affect their interaction with enzymatic systems^38,39^. The observed increase in toxicity can be interpreted according to Tweedy’s chelation theory^40,41^. As a result, antimicrobial assessments of synthesized ligands and their complexes were also performed against distinct species of bacteria (gram-negative and gram-positive).

Through its chemical implication, this work supports the different United Nations Sustainable Development Goals (UNSDGs). The catalytic degradation of harmful dyes present in water concentrates on Goal 6 which is clean water and sanitation^42^, while the antimicrobial capability of the complexes supports the goal of good health^43^. Furthermore, the development of efficient, low-cost copper catalysts promotes the sustainable industrial practices and follows the principle of SDG 12 (Responsible Production). The synthesis of new SB ligand in this work focuses on merging two biologically and catalytically active scaffolds, isatin and tryptamine, to create a multipurpose coordination framework. The extended ᴨ-system of the indole framework aids strong intermolecular ᴨ–ᴨ stacking that boosts the supramolecular assembly. Also, the electron-rich indole heterocycle enhances the nucleophilicity of the azomethine nitrogen, developing a more stable M→L coordinate bond^44,45^. Furthermore, the flexibility provided by the imine linkage allows the ligand to accommodate diverse metal ion geometry. This combination of electronic tunability and structural versatility ensures a significant improvement over rigid phenyl-based Schiff bases, providing a versatile platform for different applications^46^. Hence, in this research, a new set of isatin-based SB ligand and their metal complexes are reported, and their varied applications have been investigated. The metal complexes have facilitated the SMC reaction. They were utilized in the catalytic degradation of organic dyes, and their potential was evaluated against the different microbes to inhibit the microbial growth under ambient conditions.

## Experimental section

*Chemicals and reagents.* All the solvents employed for the study were of reagent grade. Isatin, tryptamine, copper acetate monohydrate, palladium chloride, phenylboronic acid, bromobenzene, and potassium carbonate, organic dyes used in this search, were of analytical gradient and bought from Sigma-Aldrich.

*Instrumentation.* UV-vis spectra were noted on a Shimadzu UV-2600 spectrophotometer. IR spectra were recorded on a Bruker Alpha FTIR-spectrophotometer. ^1^H NMR spectra of compounds were acquired from Bruker Avance-III HD 500 MHz spectrometers in CDCl_3_ and DMSO-d_6_. GC-MS spectra were measured on Shimadzu GCMS-QP2020. Powder X-ray diffraction (XRD) was obtained with Rigaku SMARTLAB automated diffractometer. Particle morphology was explored with a JSM-7610FPlus field emission scanning electron microscope (FESEM).

### Synthesis and characterization of Schiff base ligand (L)

**Fig. 1:**
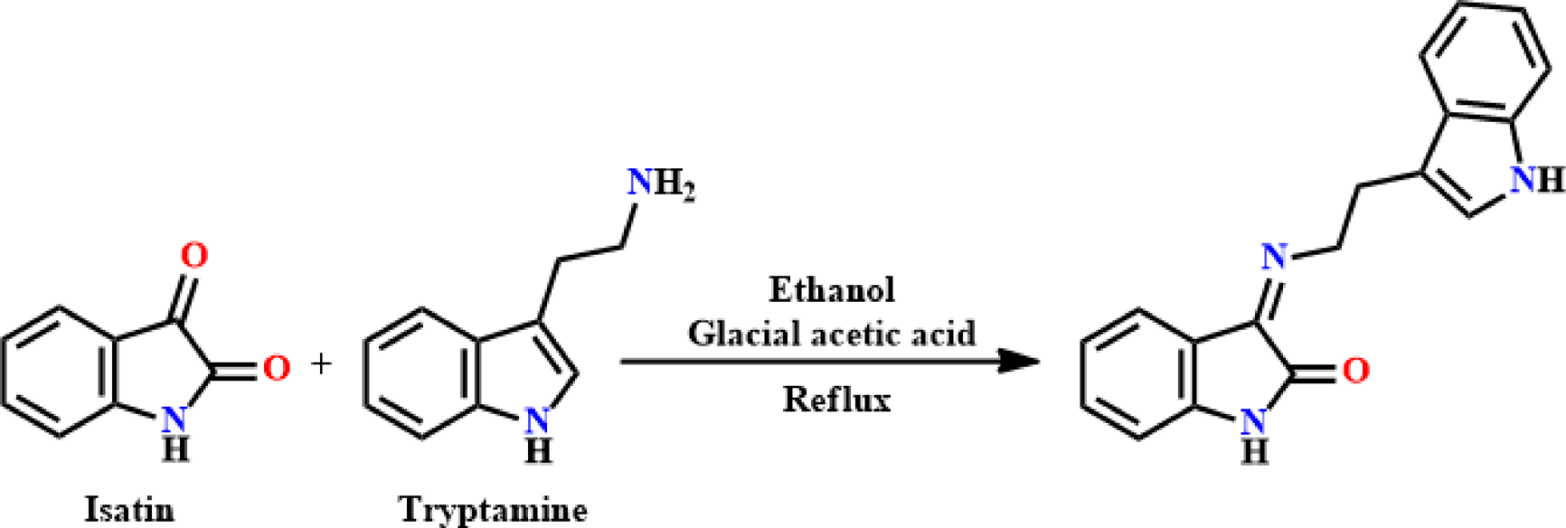
Schematic representation of SB ligand, **L** synthesis.

SB ligand (L) was prepared according to the general synthetic method by performing a condensation reaction between isatin and tryptamine (Fig. 1). Initially, 3–4 drops of glacial acetic acid (GAA) were added to stirred ethanolic solution of isatin (3.393 mmol). The formed mixture was stirred for about 30 min at ambient temperature. Subsequently, ethanolic solution of tryptamine (3.393 mmol) was mixed with the previously formed mixture, and it was refluxed for a period of 4–5 h. Excess solvent was removed using a rotary evaporator, washed with hexane, recrystallized, and dried over anhydrous CaCl_2_ in a desiccator.

L: Yield: 92%; Color: Brown. M.P.: 138 ℃. C_18_H_15_N_3_O; Mol. Wt.: 289.33; FTIR (KBr, ʋ/cm^− 1^) 1717 (ʋC = O), 1620 (ʋC = N), 1468 (Ar-C = C), 3390 (ʋNH). UV-Vis (Ethanol, λmax/nm): 225, 283. ^1^H NMR (CDCl_3_ δ ppm): 3.27–3.72 (d, 2CH_2_) 8.00–8.94.00.94 (s, NH) 7.02–7.54 (CH, benzylidenimin), 6.75 (s, -C, indole). MS (ESI; m/z): Calculated for C_18_H_15_N_3_O 289.33, Found 290.12 (M + H)^+^. Elemental Anal: Calc. (%): C-70.34, H-5.58, N-13.67; Found (%): C-69.83, H-5.41, N-14.70.

## Preparation of Schiff base copper complex

**Fig. 2:**
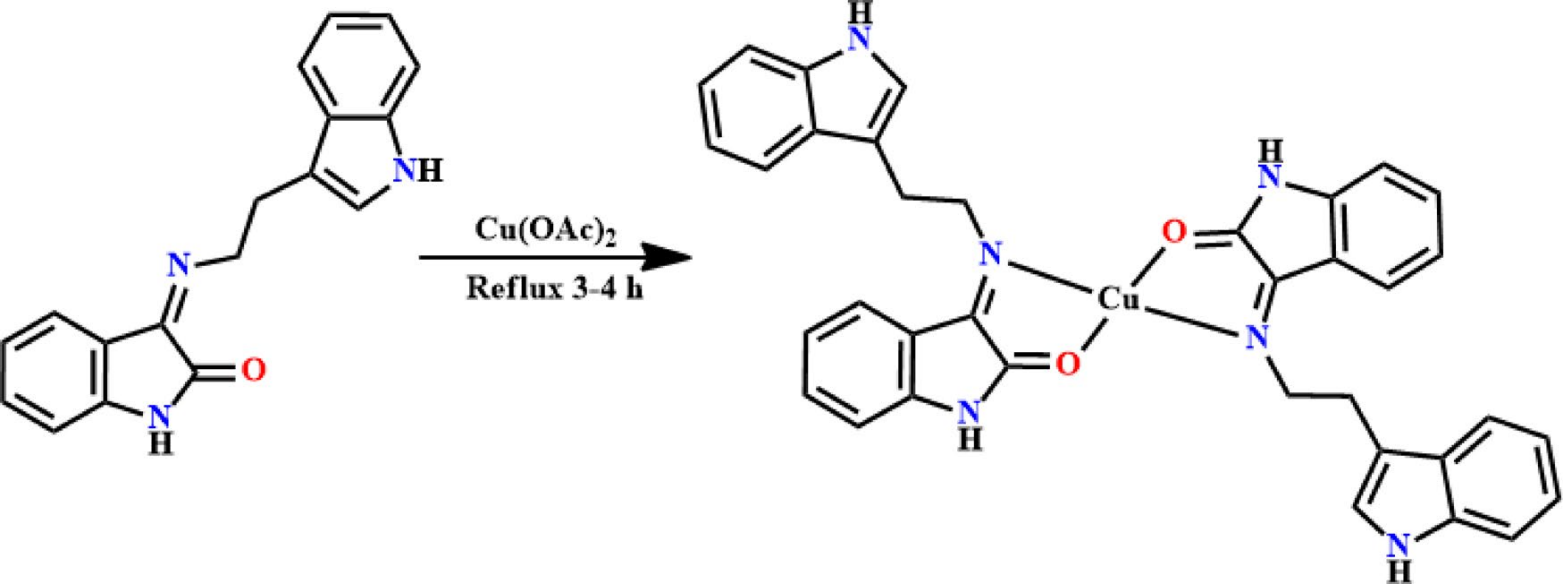
Synthesis of Copper complex of L, CuL_2_.

To a warm ethanolic solution of SB, copper acetate monohydrate dispersed in ethanol was added. The resulting mixture was refluxed for 3–4 h. Obtained copper complex was collected, rinsed with ethanol and diethyl ether, dried, and placed in desiccator over anhydrous calcium chloride (Fig. 2).

CuL_2_: Yield: 89%; Color: Maroon. FTIR (KBr, ʋ/cm^− 1^) 1625 (ʋC = O), 1504 (ʋC = N), 3436 (ʋNH). UV-Vis (Acetonitrile, λmax/nm): 240, 373, and 481. MS (ESI; m/z): Calculated for C_36_H_30_N_6_O_2_Cu 642.21, Found 637.14.

## Preparation of Schiff base palladium complex

**Fig. 3:**
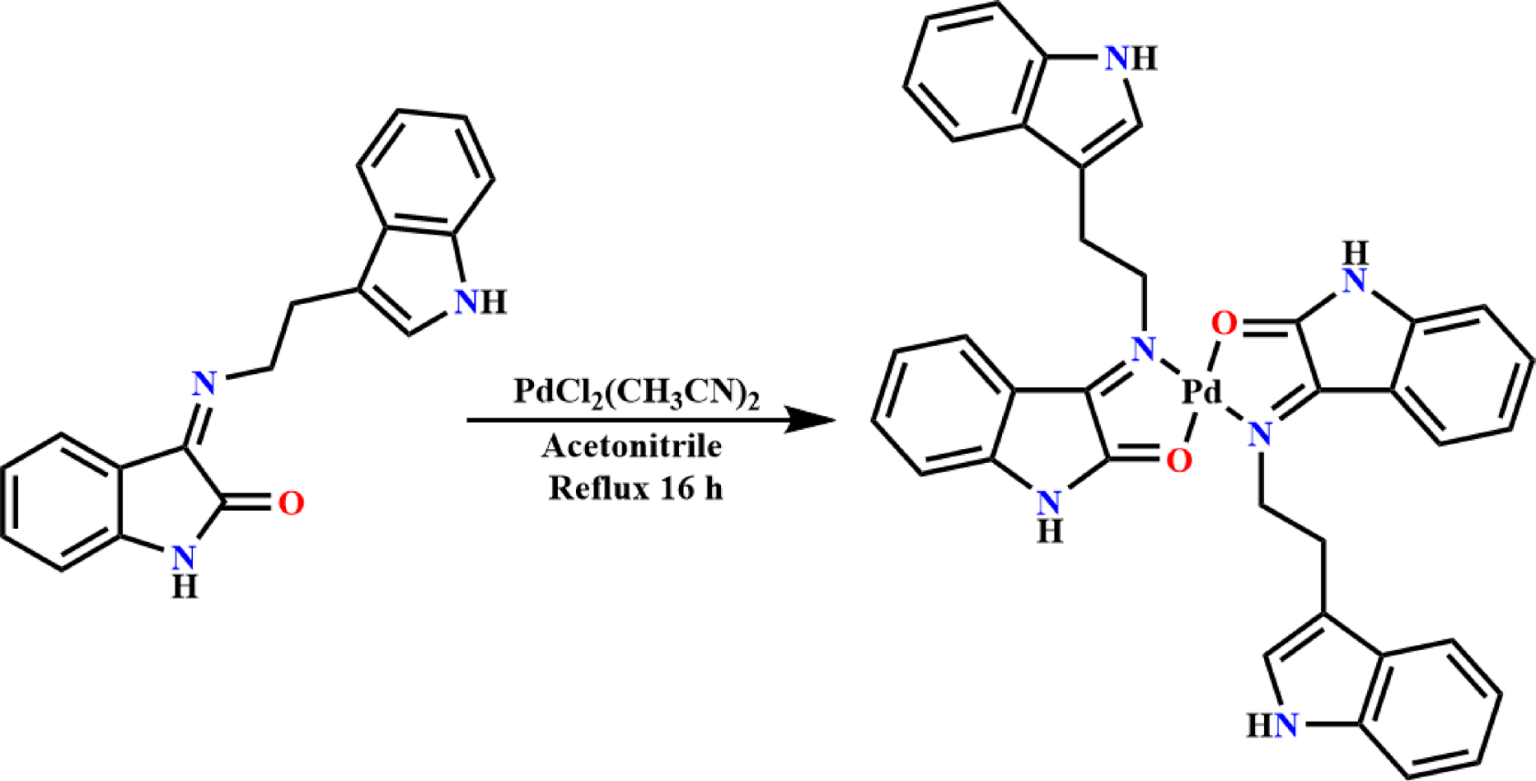
Synthesis of Palladium complex of L, PdL_2_.

Bis(acetonitrile)palladium dichloride was formed according to the standard reported procedure^47^. Subsequently, PdCl_2_(CH_3_CN)_2_ (0.350 mmol) was added to a solution of SB in acetonitrile (0.350 mmol) (Fig. 3). The resultant solution was allowed to reflux overnight. Excess solvent was removed, reduced to half of its volume, and stored in freezer for 3–4 days for the complex to crystallize. Afterwards, it was washed with diethyl ether and dried under vacuum.

PdL_2_: Yield: 61%; Color: Dark brown. FTIR (KBr, ʋ/cm^− 1^) 1685 (ʋC = O), 1532 (ʋC = N), 3664 (ʋNH). MS (ESI; m/z): Calculated for C_36_H_36_N_6_O_2_Pd 684.15, Found 683.13.

## Catalytic studies

### Carbon–carbon cross-coupling reaction

In a typical run, a mixture of bromobenzene (2.0 mmol) and phenylboronic acid (3.0 mmol) was placed in a reaction vial, followed by addition of a copper and palladium complex (5 mol %) and K_2_CO_3_ (4.0 mmol) with DMF-H_2_O (10 ml). The resulting mixture was heated for 4 h at 80 ℃. The reaction progress was observed using TLC. Upon completion of reaction, the solution was cooled to room temperature. The product was then separated using hexane, and the resulting organic layer was removed and dried using anhyd. Na_2_SO_4_. Biphenyl was obtained as a product due to the homocoupling of phenylboronic acid, as evidenced from GCMS data.

### Catalytic degradation of organic dyes

For this study, the catalytic utility of synthesized copper complexes was explored in specific conditions. Attempts to develop easily produced, efficient, and reusable catalysts are ongoing. The catalytic assessment of the chemically derived SB copper complex was evaluated for degradation of aq. MO, MEB, Rh-B, and CR dyes. Sodium borohydride was used as a reductant^48^. As a negative constraint, the reaction was examined using only NaBH_4_ in the absence of any catalyst. For all dye systems, the degradation reactions were conducted with equal quantities of dye, reductant, and catalyst in separate experimental sets. In a usual practice of degradation, an aqueous solution of dye (100 mg L^− 1^, 25 ml) was mixed with a freshly prepared. The period of whole decolorization of each dye was recorded to assess the catalytic activity.

## Evaluation of antibacterial activity

Antibacterial potential of the above compounds was evaluated against the bacterial strains *Escherichia coli* (MTCC-443), *Pseudomonas syringae* (MTCC-1604), *Staphylococcus aureus* (MTCC-96), and *Staphylococcus epidermidis* (MTCC-435). Streptomycin was used as positive control for antibacterial activity. The test organisms were subcultured in nutrient broth for 24 h at 37 °C. 25 mL of sterilized medium was poured into sterile petri plates and allowed to coagulate. Inoculation was conducted using a micropipette with sterilized tips, and 100 µL of every activated bacterial strain was spread uniformly over the agar surface. Subsequently, 100 µL of the test compound suspension was introduced into each well, while dimethyl sulfoxide (DMSO) served as the negative control. After incubating all plates for one day at 37 °C, the growth inhibition zones (in mm) was then calculated to assess antibacterial efficacy.

## Evaluation of antifungal activity

Likewise, the antifungal potential of the compounds was evaluated against fungal strains: *Aspergillus flavus* (MTCC-1973) and *Candida albicans* (MTCC-854). The cultures were prepared using malt extract medium for *A. flavus* and malt yeast extract medium for *C. albicans*. Dimethyl sulfoxide (DMSO) was used as negative control. Like antibacterial test, 25 mL of treated medium was poured onto sterile plates and allowed to coagulate. Inoculation was done with a micropipette using pre-sterilized tips, and 100 µL of activated fungal spore suspension was evenly spread over the agar surface. Subsequently, 100 µL of the test compound suspension was introduced into each well. Later, the plates were placed in incubator at 25 °C for 48 h, and the growth inhibition zones were calculated (mm).

## Results and discussion

**Fig. 4:**
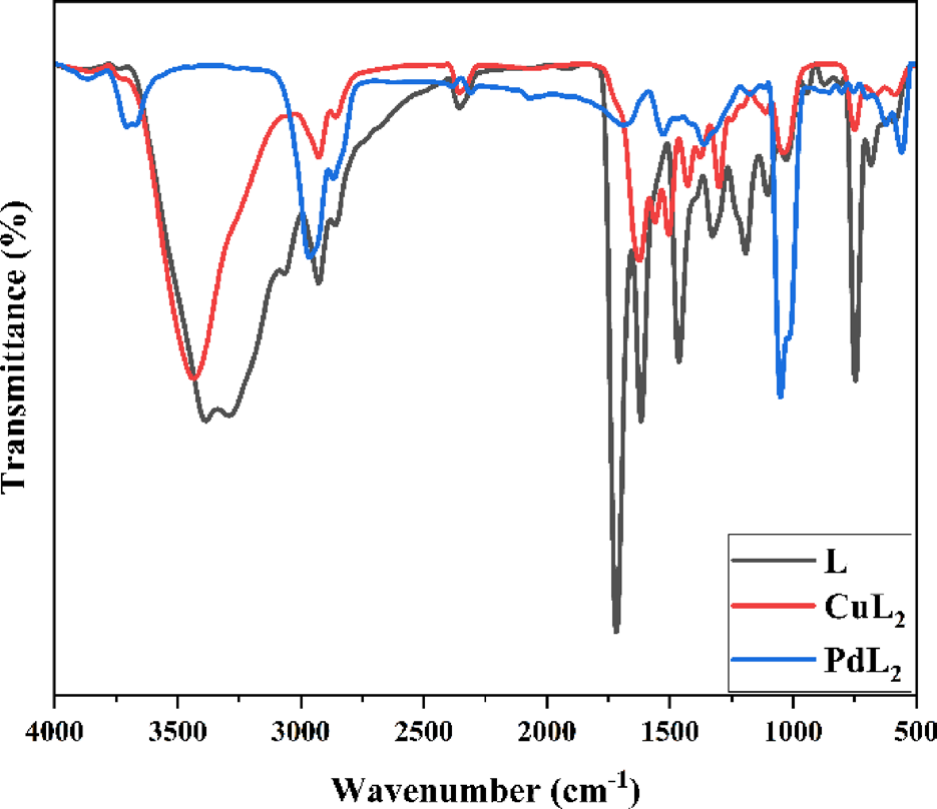
FTIR spectra of L, copper and palladium complex.

*FTIR spectroscopic*. Studies Presence of different functional groups in SB ligand, Cu and Pd complexes were detected with the help of infrared spectra (Fig. 4). There are three donor sites available in ligand which are the isatin oxygen, isatin nitrogen, and the azomethine group. A medium band appearing at 3390 cm^− 1^ in spectra of the ligand was ascribed to the N-H stretching^49^. This band disappears in spectra of complexes. The infrared band of free SB displayed a strong band in 1620 cm^− 1^ region, which is representative of the azomethine group. While in complexes, this band moved to a lower frequency, indicating its involvement in chelation with metal ion. The strong band appearing at 1717 cm^− 1^ was ascribed to ν(C = O) band in the spectrum of the ligand^50^.

**Fig. 5:**
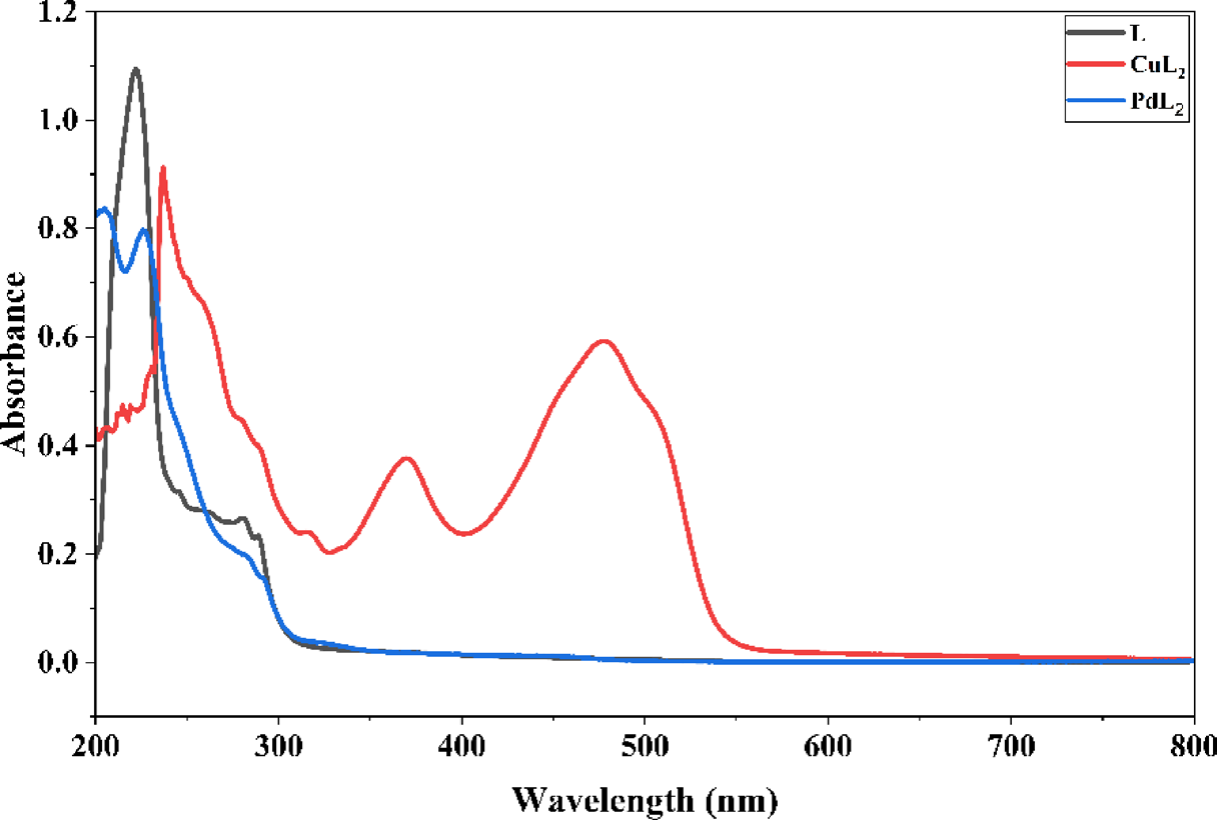
UV-vis spectra of L, copper and palladium complexes.

*UV-vis spectroscopy.* Electronic absorption spectra of synthesized L and complexes were taken in ethanol and acetonitrile, respectively (Fig. 5). The SB displayed strong bands at 283 and 225 nm owing to n→π* and π→π* transition of ligand. On complexation, the n→π* absorption band is slightly shifted (minor bathochromic shift) but remains prominent in UV region, suggesting that azomethine nitrogen engaged in chelation. The spectra of copper complex demonstrated three different absorption band at 240, 373, and 481 nm. Appearance of an additional intense broad band in the visible area at 481 nm relates to d-d transition. This feature, along with the shifting of the azomethine IR band to a lower frequency (1504 cm^− 1^), supports a distorted square planar geometry. The broadening of the visible band is a standard indication of Jahn-Teller distortion, where the d^9^ electronic configuration of Cu^2+^ leads to a loss of perfect octahedral symmetry, typically resulting in axial elongation to lower the energy of the system^51^. The band seen at 373 nm may be ascribed to LMCT (ligand to metal charge transfer), indicating conjugation of metal-ligand bonding^52^. While the palladium complex exhibits absorption bands at 205, 227, and 284 nm and does not show any band in the visible area. The absence of absorption bands in the visible region of Pd complex, combined with prominent UV transitions at 205, 227, and 284 nm, is consistent with a quadrilateral (square planar) nature. This planar geometry was further confirmed by the IR data, that shows the participation of N, O-donor sites, exclusively the shift of the carbonyl (C = O) and azomethine (C = N) groups, indicating a stable 1:2 metal-to-ligand stoichiometry that supports the highly symmetrical planar arrangement.

**Fig. 6:**
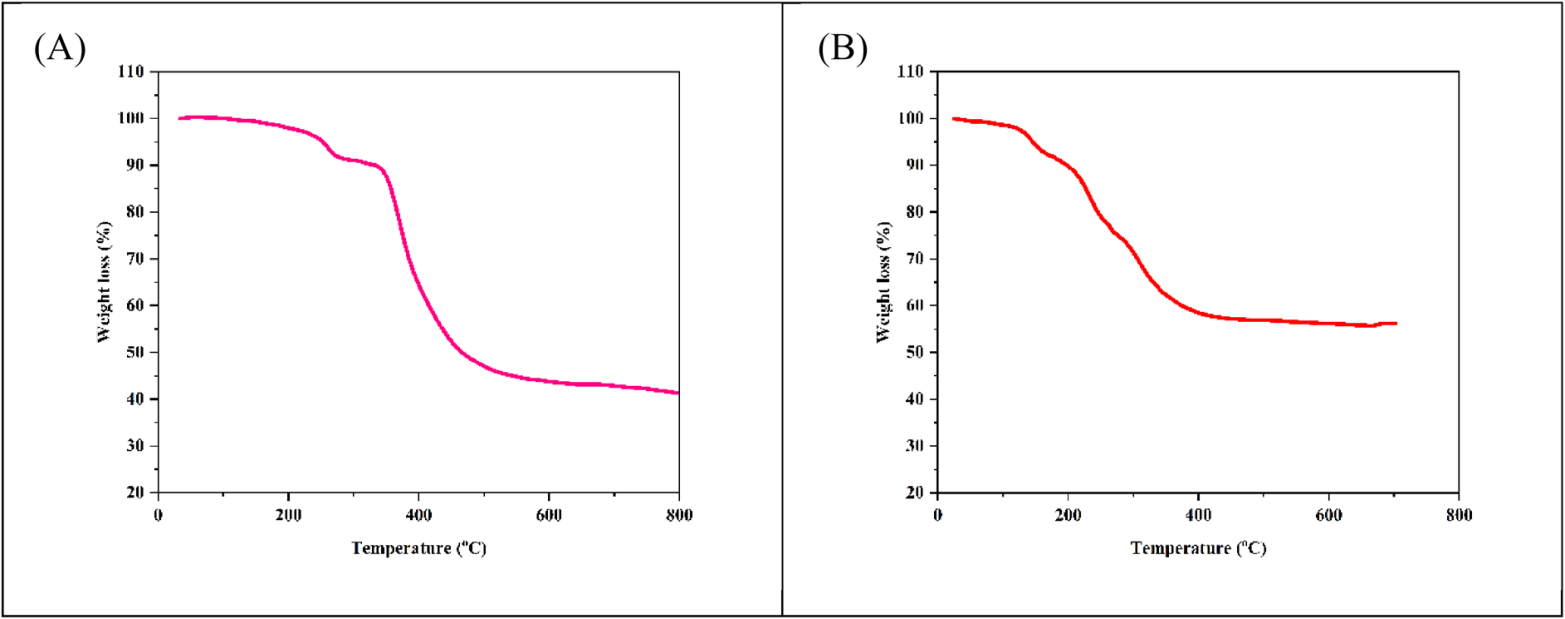
TGA curve of copper (A) and palladium (B) complex.

*Thermal decomposition.* Thermal analysis provides the thermal strength and decomposition behavior of compounds. Also, it is useful for obtaining valuable data about their structural characteristics and the preferential coordination of distinct groups directly bound to metal ions (Fig. 6)^53^. The thermal decomposition pattern of the complexes was determined using TGA curve. Cu-complex was stable up to 250 ℃ and its breakdown began at this temperature. The first observable mass loss, typically about 250 °C corresponds to the vaporization of physically adsorbed water molecules or loosely bound molecules and volatile components. The next major decline from 350 to 500 ℃ corresponds to the thermal degradation of ligand moiety bonded to the metal. In the decomposition manner of the Cu(II) complex, mass losses occur with three phases. It is verified with three exothermic peaks in the thermogram of Cu-complex. In case of the Pd-complex, the initial mass loss was seen about 150 °C, corresponds to the evaporation of immersed water or solvent molecules. Hereafter, the weight remained unchanging up to 340 °C, indicating high thermal stability before the breakdown of organic components started. Afterwards, the next decline related to the decomposition of SB ligand itself, leaving metal oxide as a product^54^.

**Fig. 7:**
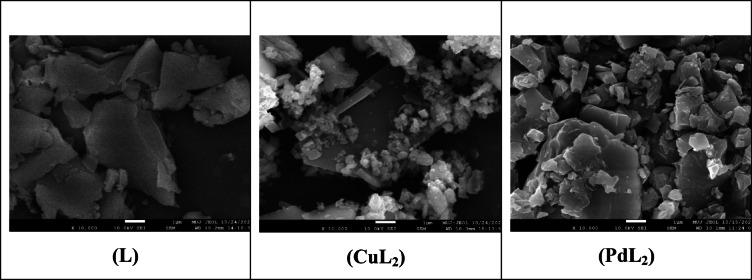
FESEM micrograph of L, Cu and Pd complex.

*FESEM analysis.* Field Emission Scanning Electron Microscopy investigation provides critical understandings into the external morphology, structural features, and particle size of compounds. The FESEM analysis of SB and its copper and palladium complexes are given in Fig. 7. FESEM images of the pure SB ligand often reveal a smooth, homogeneous surface with irregular shapes, indicating an amorphous or partially crystalline nature. When this SB coordinated with copper(II) and palladium (II) ions, the FESEM micrographs showed prominent morphological changes, such as increased aggregation and crystalline domains, and led to the formation of dense clusters^55^. In comparison to the ligand, metal complex has a rougher surface and porous texture, reflecting enhanced intermolecular interactions and packing density induced by metal chelation. These changes confirm successful complexation and suggest improved surface area, which can enhance catalytic and biological activity^56^.

**Fig. 8:**
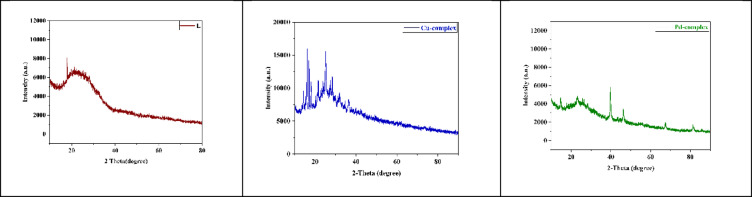
X-ray diffraction (XRD) patterns of L, Cu and Pd complex.

*X-Ray diffraction analysis.* In this analysis, the XRD method was utilized to discover the crystal parameters of synthesized compounds and were illustrated in Fig. 8. Obtained data shows the 2θ value and relative intensity of synthesized complexes differ from ligand. The L demonstrates a broad peak at ~ 2θ = 22.19°, indicating its largely amorphous nature^57^. However, both the complexes show sharp peaks, indicating their crystalline nature. Cu-complex displays three sharp peaks at 2θ = 16°, 17.15°, and 25.21° and some other peaks also at different 2θ value, suggesting its highly crystalline character. In contrast, Pd-complex exhibits a sharp major peak at 2θ = 39.74° and other small peaks also. These outcomes indicated that ligand was effectively coordinated with metal ions to form complexes.

The synthesized metal complexes consists of crystallites, wherein the crystallite size (D) can be determined using the *Debye–Scherrer* (DS) equation:

$$\:\mathrm{D}=\frac{K\lambda\:}{\beta\:cos\theta\:}$$

where, β represents the full width at half maximum (FWHM) of the diffraction peak, λ is the wavelength of X-ray used (1.5406 Å), k is a constant (0.9), and θ is the Bragg diffraction angle. The crystallite sizes were calculated for each complex using the most intense 2θ peak, and the results are summarized in Table 1. The copper complex exhibits crystallite sizes ranging from ~ 11.13 to 37.04 nm, confirming its nanocrystalline nature. However, the palladium complex displays significantly smaller crystallite sizes, ranging from ~ 2.36 to 10.59 nm. These values were observed to match with the ones reported for similar complexes^58^.

**Table 1:** Particle size of complexes calculated by Debye-Scherrer equation (DS).

Compound	2θ	FWHM (°)	D (nm)
CuL_2_	16.124	0.3654	21.94959
PdL_2_	39.806	0.7973	10.59214

*Bioactivity studies*. All the synthesized compounds, including SB and transition metal complexes, were tested for their antimicrobial activity (antibacterial and antifungal). The results of the investigation were presented in Table 2 in the form of their zone of inhibition.

**Table 2:** Antimicrobial study results of synthesized compounds.

Compounds	*S. aureus*	*S. epidermidis*	E. coli	*P. syringae*	*C. albicans*
	Zone of inhibition (mm)				
L	5	7	-	-	10
CuL_2_	11	12	19	8	13

### Antibacterial activity

SB and its metal complexes were assessed for antibacterial action employing the agar well diffusion method. The evaluation was conducted against four pathogenic bacterial species, involving gram-positive (*Staphylococcus aureus* and *Staphylococcus epidermidis*) and gram-negative species (*Escherichia coli* and *Pseudomonas syringae*). Out of the different tested compounds, L is positively showing activity towards *S. aureus* and *S. epidermis* only, while completely inactive against others. Notably, Pd-complex do not show any positive activity towards all the tested pathogen; thus, it is completely ineffective with respect to its antibacterial action. In contrary, Cu-complex showed positive results towards all the tested organisms and showed the highest zone of inhibition with respect to *S. epidermis*. Precisely, after chelation with copper ions, the complexes were more effective than the standard antimicrobial drug, Streptomycin, which had an inhibition zone of 15 mm for *E. coli.* The increased activity of complex is owing to the chelation factor^59^. Chelation mainly decreases the polarity of metal ion thereby increasing the lipophilicity of complex. This helps diffusion across the lipid-rich bacterial membrane more capably than free ligand. Also, coordination can reduce the overall positive charge on metal center, thus stabilizing the complexes and improving their ability to interact with negatively charged bacterial surfaces. The enhancement in activity was interpreted through Tweedy’s Chelation Theory. Upon coordination, the positive charge of the metal ion was partially shared with the donor atoms (N, O) of the ligand, thereby leading to ᴨ-electron delocalization over the entire chelate ring. This significantly reduces the polarity of the metal ion. The reduction in polarity increases the lipophilicity of the complex, allowing it to penetrate the lipid membranes of the bacterial cell wall more effectively (Overtone’s Concept). According to Overtone’s Concept, this enhanced liposolubility allows the complex to reach intracellular targets more efficiently, where it may deactivate essential cellular enzymes or disrupt the respiratory chain of the microorganisms. Thus the metal complex could interfere with the normal metabolic processes or bind to DNA/proteins more efficiently than the free ligand.

### Antifungal activity

By employing identical procedures, the antifungal potential of SB and the metal complexes was checked against two fungal types, i.e., *Aspergillus flavus* and *Candida albicans*. Both ligand and Cu-complex is active towards *C. albicans* with complex having high antifungal potential with respect to its corresponding ligand. None of the tested compounds were active against *A. flavus*. The varying activity against the fungus might be due to differences in their cell wall composition. *C. albicans* comparably has a thinner and more porous cell, allocating easier access for lipophilic SB and complexes^60^.

### Comparisons with the results reported for antibacterial activity using Schiff base copper complexes

Current investigation proved that the antimicrobial efficiency of SB metal complexes consistently exceeds their parent ligands. The enhancement is largely ascribed to Tweedy’s Chelation Theory, where the coordination of metal ions reduces the polarity of the ligand, thereby increasing the lipophilicity of complexes, facilitating their migration through the lipid membrane of microbe. Below is a brief comparison of the results reported for antimicrobial activity in recent studies (Table 3**)**.

**Table 3:** Comparative study of antimicrobial activity against bacterial and fungal strains.

Compounds	*E. coli*	*S. aureus*	*P. aeruginosa*	*C. albicans*	References
	Zone of inhibition (mm)				
Cu-complex	10	10	8	-	^61^
Cu-complexes	-	-	10–18	3–18	^62^
Cu-complex	13	14	-	0	^63^
Cu-complex	-	16	14	-	^64^
Cu-complex	19	11	8	13	This work

**Fig. 9:**
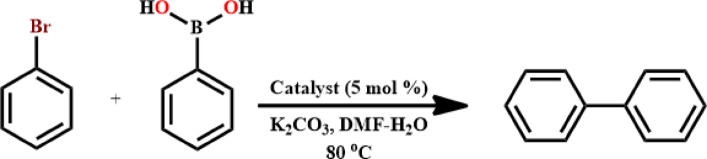
Catalytic C-C bond forming reaction of phenyl boronic acid and bromobenzene.

*Catalytic cross-coupling studies.* The synthesized copper and palladium complexes could facilitate an SMC reaction, under catalytic conditions. Primarily, the reaction was performed between bromobenzene and phenylboronic acid as standard substrates (Fig. 9). Various reaction parameters like the amount of catalyst, solvent, base, time, and temperature were optimized and analyzed. Among the different solvents (acetonitrile, acetone, DMF-H_2_O, 1,4-dioxane, and toluene), highest catalytic yield was seen with DMF-H_2_O. Also, the use of base is an important factor for SMC reaction. In this catalytic cycle, K_2_CO_3_ was found to be the best one. Different amounts of catalyst loading from 1 mol % to 5 mol %, were optimized. The dependency of product yield on reaction time for the cross coupling was tested by analyzing the reaction mixture at usual intervals of time under the same condition. Cu-complex does not show any prominent result within 4 h, but an increase in yield of the product was found when the time increases to 16 h. In contrast, Pd-complex gave maximum yield with 4 h of reflux. Here, for the reaction between phenylboronic acid and bromobenzene, reaction gave 15% and almost 100% yield of the desired product with Cu and Pd complexes respectively, at 5 mol % catalyst loading. Moreover, the catalytic reaction was also tested in the presence of SB as a catalyst; however, no successful yield of cross-coupled product was obtained.

**Table 4:** Catalytic Dye Degradation results with synthesized complex.

Catalyst	Base	Solvent	Temperature (℃)	Time (h)	Conversion (%)	TON; TOF (h^− 1^)
Pd-complex	K_2_CO_3_	DMF-H_2_O	80	4	~ 100	20; 5
Cu-complex	K_2_CO_3_	DMF-H_2_O	80	4	No conversion	-
Cu-complex	K_2_CO_3_	DMF-H_2_O	80	16	15	3; 0.188

Reaction conditions: bromobenzene (2 mmol), phenylboronic acid (3 mmol), K_2_CO_3_ (4mmol), solvent (5 mL), 16 h, catalyst (5 mol %).

The catalytic performance of the synthesized Cu(II) and Pd(II) complexes in SMC reaction was basically related to the ligand’s electronic properties, the metal’s oxidation state, and the coordination geometry. The isatin-tryptamine SB presents a stable N, O-donor structure, with strong σ-donation from the azomethine nitrogen and coordination with the isatin oxygen to anchor the metal centers. The Pd(II) complex, which acts as an efficacious pre-catalyst, enables in situ reduction to the active Pd(0) species for the catalytic cycle. On the contrary, the Cu(II) complex shows a distinctive broad d-d transition band at 481 nm, which was the indicative of d^9^ Jahn-Teller distortion and a more flexible coordination sphere. This axial flexibility in the Cu environment likely aids the coordination of the phenylboronic acid and the base, endorsing the coupling reaction even with a non-precious metal center. Further, both complexes demonstrated high thermal stability (CuL_2_ up to 250 °C and PdL_2_ up to 340 °C), which ensures that the metal complexes retained their stability under reflux conditions for biphenyl formation.

*Comparison with reported Pd-Schiff base complexes for catalytic C–C cross coupling reactions.* Recent analysis verified that the catalytic efficiency of SB metal complexes constantly exceeds their parent ligands. The enhancement is largely ascribed to the metal coordination which increases the stability of complexes. The distinctive steric and electronic environment present across the metal centre expressively impacts catalytic activity. Below is a brief comparison of the results reported for catalytic activity in literature (Table 5).

**Table 5:** Comparative study of catalytic activity in Suzuki-Miyaura reaction.

Pd-complexes	Catalytic reaction conditions	Reactant	Yield (%)	Reference
	KOH, EtOH, 70 ℃, 5 h	Aryl halide and phenylboronic acid	47–99%	^65^
	K_2_CO_3_, PEG-400, 100 ℃, 10 h	Aryl halide and phenylboronic acid	86–97%	^66^
	K_2_CO_3_, H_2_O, 80 ℃, 4 h	Aryl chloride and phenylboronic acid	66–98%	^67^
PdL_2_	K_2_CO_3_, DMF-H_2_O, 80 ℃, 4 h	Bromobenzene and phenylboronic acid	99%	This work

**Fig. 10:**
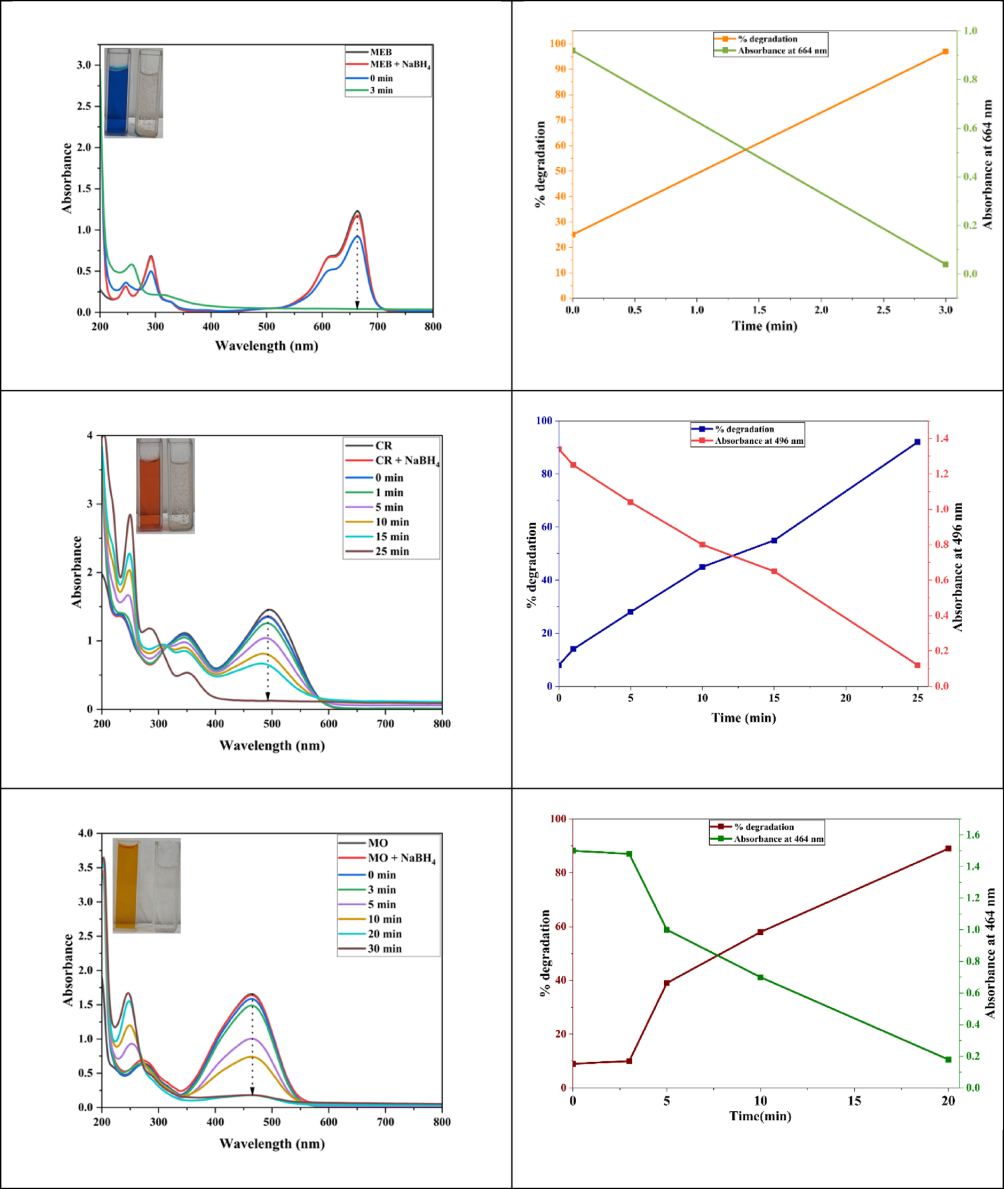

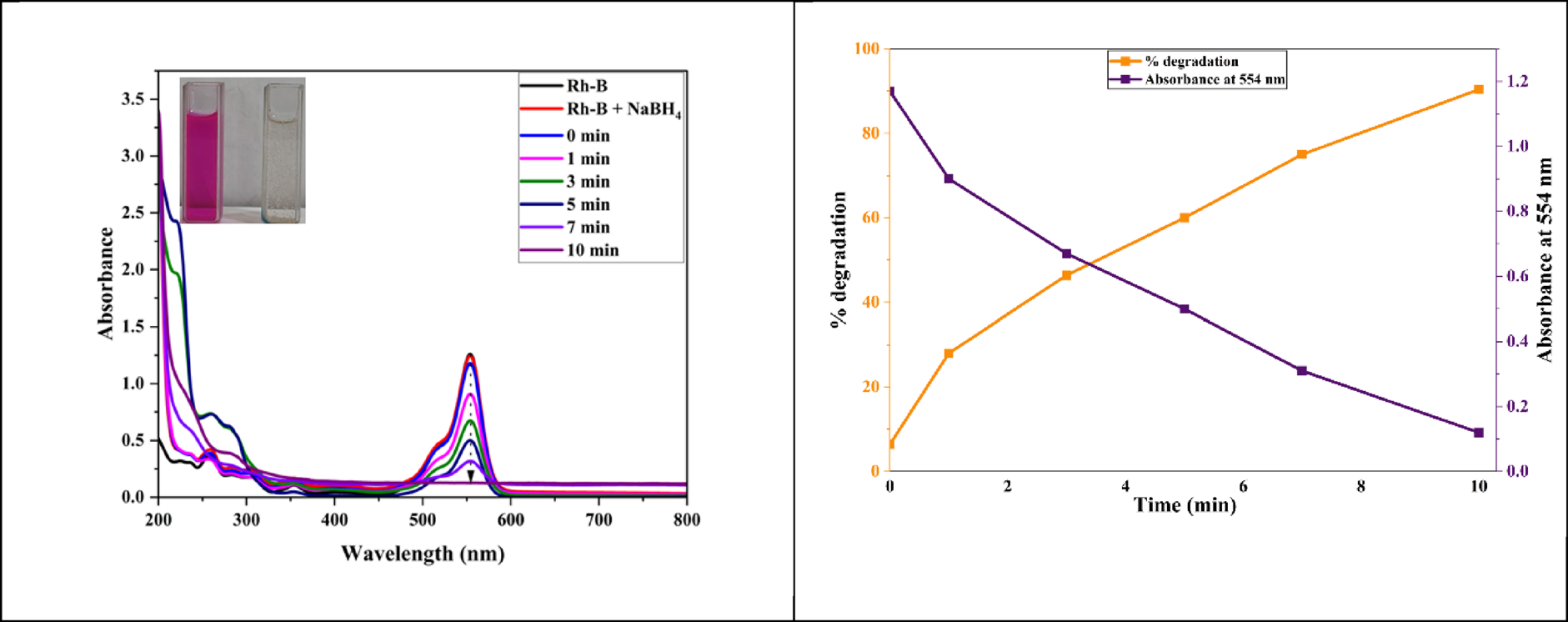
Time-dependent absorption spectra of organic dyes, catalyzed by CuL_2_.

**Fig. 11:**
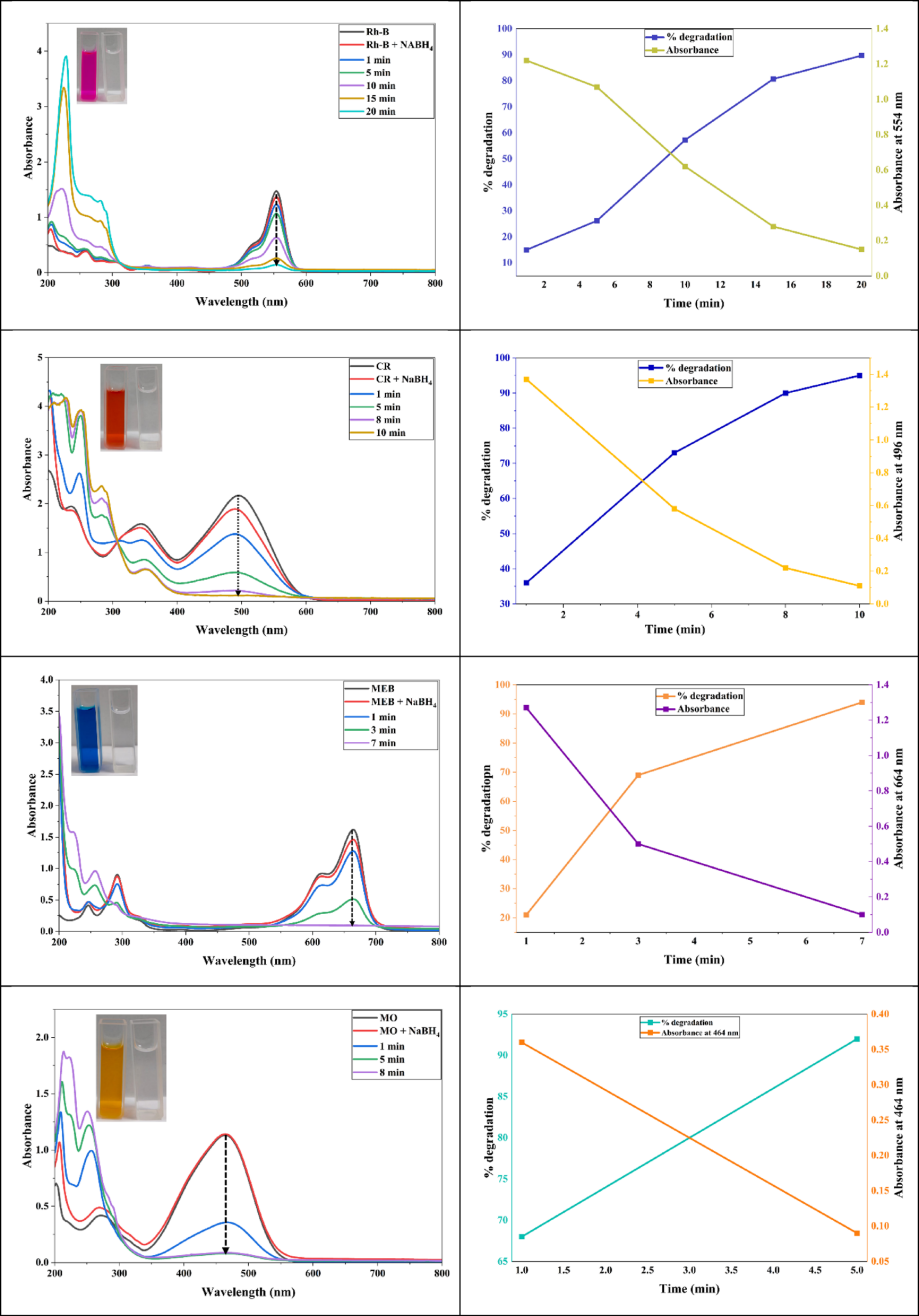
Time-dependent absorption spectra of organic dyes, catalyzed by PdL_2_.

*Catalytic dye degradation studies*. The catalytic efficiency of modified SB complex for the reduction of textile dyes, specifically MO, Rh-B, MEB, and CR, was scientifically investigated via time-resolved experiments using sodium borohydride (NaBH_4_) as the reducing agent. (Table 6) Initially, control experiments were performed by subjecting individual dye solutions (25 ml, 100 mg L⁻¹) to NaBH_4_ in the absence of any catalyst to evaluate the basic reducing ability of NaBH_4_ alone. UV-visible spectrophotometric analysis showed that no noticeable reduction or degradation occurred for any of the dye molecules under these conditions, confirming that NaBH_4_ is ineffective in catalyzing dye degradation without an initiator. Subsequently, 5 mg of the modified SB copper and palladium complex was introduced separately into each dye solution containing of NaBH_4_. Upon addition of the complexes, a prominent catalytic activity was noted, as indicated by rapid color loss and reduced absorbance peaks recorded in UV-vis spectra. For MEB, the adsorption bands were monitored at 664 nm and the system exhibited complete degradation and decolorization within just 3 min for Cu-complex, highlighting the complex’s high catalytic proficiency. The conversion results were found to be 97% in 3 min, while the palladium complex degrade MEB within 7 min with the conversion of 94%. In case of MO, the absorption band was monitored at 464 nm and the copper complex-initiated degradation within 1 min, with total decolorization attained in 30 min. The degradation results were achieved as 10, 39, 58, and 89% at 3, 5, 10, and 20 min, respectively. Also, the palladium complex started degradation within 1 min and complete decolorization attained in 5 min. The degradation percentage were achieved as 68 and 92% at 1 and 5 min respectively. CR followed a similar path, displaying onset of degradation within 1 min (at 496 nm) with Cu-complex and complete breakdown after 25 min with the percent conversion of 92%. However, Pd-complex started degrading the CR within 1 min and the total breakdown was achieved after 10 min with 95%. For Rh-B, the adsorption bands were monitored at 554 nm and the full degradation occurred in 10 min and the solution turned completely colorless thereafter. The reduction conversions with copper catalyst were attained as 28, 46.4, 60, 75, and 90.4% at 1, 3, 5, 7, and 10 min respectively. Whereas, the conversion in presence of palladium catalyst were attained as 15, 26, 57, 81, and 90% at 1, 5, 10, 15, and 20 min respectively. These comparative results provide constraining evidence for the considerable role of the SB copper and palladium complexes in assisting electron transfer from NaBH_4_ to dye molecules, thereby accelerating their reductive breakdown. The marked acceleration of dye degradation relative to mere NaBH_4_ treatment demonstrates the efficacy of the metal catalyst, likely attributable to its ability to activate NaBH_4_ and facilitate dye reduction via enhanced surface interactions and improved charge transfer kinetics. The experiment highlights the potential environmental and practical benefits of using synthesized transition metal complexes for efficient dye remediation, further confirmed by the spectrophotometric data presented in Figs. 10 and 11. While UV-Vis monitors the disappearance of the characteristic absorption peak at particular value of λ max for different dye molecules, it was also observed the simultaneous decrease of peaks in the UV region, which corresponds to the breakdown of different functional groups into smaller components. Furthermore, the mechanism may be rationalized based on previous studies using SB metal complexes^68^. Initially, NaBH_4_ generates hydride ions (H⁻), which transfer electrons to the metal center in the complex, forming an active metal-hydride species (M-H). This species interacts with dye molecules via coordination to the imine nitrogen, facilitating hydride transfer to the dye’s chromophore, leading to bond cleavage and colorless products. Often formation of small organic acids and eventual mineralization (CO_2_ and H_2_O) is observed. The hypsochromic shift and the steady decrease in the absorbance at λ max suggest the fragmentation of the conjugated chromophore system. It can be presumed that the electrons target the –N=N– double bond, leading to its reductive cleavage into smaller, colorless amine fragments. Consequently, the reusability of the copper and palladium complexes were assessed in the reductive dye degradation reaction. Both catalysts were found to actively degrade the dye molecules upto three cycles, after which the time taken for degradation gradually increased.

**Table 6:** Catalytic Dye Degradation results with synthesized copper and palladium complexes.

Catalyst	Pollutants	Monitoring λ_max_ (nm)	Time (min)	Degradation (%)
Cu-complex	MEB	664	3	97.0
	MO	464	30	89.0
	CR	497	25	92.0
	Rh-B	554	10	90.4
Pd-complex	MEB	664	7	94.0
	MO	464	8	92.0
	CR	497	10	95.0
	Rh-B	554	20	90.0

## Conclusion

The present study reveals the synthesis of a new Schiff base (SB) ligand based on isatin moiety via condensation reaction of tryptamine and isatin. Likewise, metal complexes of Cu and Pd were synthesized from newly synthesized ligand. All the prepared compounds were characterized by numerous spectroscopic methods comprising UV-vis, FTIR, and ^1^H NMR spectroscopy. Moreover, the structural features of the compounds were also studied with the help of XRD, FESEM, EDAX, and TGA analysis. The effective synthesis and characterization of these compounds, aided by analytical and spectroscopic data, supports their structural stability and coordination behavior towards metal ions. The compounds were screened for antimicrobial testing, in which only copper complex gave positive results towards antibacterial action, while the palladium one is completely ineffective. These findings point out that SB complexes contribute to the global effort to develop new therapeutic agents to combat antibiotic-resistant bacteria, aligning with good health to end epidemics of communicable diseases. For both the metal complexes, the catalytic efficiency was investigated in the degradation/reduction of organic contaminants existing in water (MEB, MO, CR, and Rh-B) at ambient temperature. However, the highest degradation percentages were obtained in the order of MEB > Rh-B > CR > MO within 3, 10, 20, and 25 min separately for copper complex, while the highest degradation percentages were obtained in order of CR > MEB > MO > Rh-B within 10, 7, 8, and 20 min for palladium complex. This emphasizes their potential as effective catalyst towards wastewater treatment and contributes to sustainable environmental remediation. The study demonstrates the high efficiency of the Cu SB complex in the reductive degradation of organic dyes. By providing a pathway to remove toxic industrial effluents from water bodies, this research directly supports Clean Water and Sanitation goal of UNSDG, which aims to improve water quality by reducing pollution and minimizing the release of hazardous chemicals into the open water sources. Additionally, the catalytic functioning of these compounds in SMC reaction was monitored under different conditions. Among the Cu and Pd complexes, PdL_2_ was seen as highly active and efficient catalyst with better catalytic proficiency and gave approximately 100% yield of the desired biphenyl product. In contrast, CuL_2_ displays very little activity towards the biphenyl formation (15%). This highlights their ability to mediate key C-C bond formation under mild conditions. Furthermore, the efficiency of these complexes in SMC reactions promotes “Green Chemistry” by reducing waste, reagents required and energy consumption in the synthesis of complex organic molecules and pharmaceuticals. Beyond the current study, the flexibility of this ligand system attracts further investigation with other transition metals such as nickel (Ni), cobalt (Co), iron (Fe), and ruthenium (Ru). The presence of N and O coordination sites within the isatin moiety suggests that Ni and Co complexes would serve as effective, earth-abundant catalysts for electrocatalysis, in CO_2_ activation or functionalization as well as in medicinal chemistry. Also, the structural similarity of the tryptamine-isatin framework to biologically active compounds enable these complexes, particularly with Pd and Ru, to be promising candidates for anticancer research. These complexes would exhibit enhanced DNA-binding affinity or targeted cytotoxicity. These opportunities represent promising directions for future research to expand the scope of this versatile coordination system.

## Data Availability

All data supporting the findings of this study are available within the Main text of the paper.
